# Health literacy and self-management among middle-aged and young hypertensive patients: a parallel mediation effect of illness perception and self-efficacy

**DOI:** 10.3389/fpsyg.2024.1349451

**Published:** 2024-05-03

**Authors:** Yaqing Liu, Feng Jiang, Meicheng Zhang, Haoran Niu, Jianbo Cao, Sixian Du, Hongfeng Chen, Hui Wang, Liwen Gong, Feifei Rao, Huan Wu

**Affiliations:** School of Medicine and Health Management, Tongji Medical College, Huazhong University of Science and Technology, Wuhan, China

**Keywords:** health literacy, self-management, illness perception, self-efficacy, middle-aged and young hypertensive patients

## Abstract

**Background:**

Hypertension is increasingly prevalent among young and middle-aged populations in rural China, accompanied by suboptimal self-management. Given that this population forms the backbone of the labor force, enhancing their self-management capabilities is crucial for improving overall population health. Studies indicate that individuals with good health literacy are more likely to effectively manage their health.

**Methods:**

Grounded in the health literacy skills framework, a model was constructed in this study to examine the impact of health literacy on self-management among young and middle-aged hypertensive patients in rural China. Meanwhile, the mediating roles of illness perception and self-efficacy were also verified. Using a multi-stage stratified random sampling method, 338 patients were recruited to participate in the study. Structural equation modeling was utilized to establish the relationship model, and bootstrap tests were carried out to examine the mediating effects.

**Results:**

The average self-management score was 70.45 ± 11.36. Health literacy exhibited a positive correlation with self-management (standardized *β* = 0.372, *p* < 0.001). The mediating effects through illness perception and self-efficacy were 0.040 and 0.236, constituting 6.68 and 39.31% of the total effect, respectively.

**Conclusion:**

Illness perception and self-efficacy serve as parallel mediators amid the association between health literacy and self-management. Implementing psychological counseling and health education is imperative for augmenting self-management competence and cultivating an adaptive coping mentality.

## 1 Introduction

As accentuated in the “China Cardiovascular Health and Disease Report 2021,” hypertension constitutes a salient risk factor precipitating cerebrovascular disease (Report on Cardiovascular Health and Diseases in China 2021, 2022). The hypertensive population has burgeoned to an overwhelming 245 million in China, with a ceaselessly elevating prevalence, especially conspicuous in rural locales. To compound concerns, hypertension is now more prevalent among young and middle-aged demographics, culminating in a significant increase in incidence (Zhang et al., 2021).

Self-management is defined as the individual’s ability to manage the symptoms, treatment, physical and psychosocial consequences, and lifestyle changes inherent in living with a chronic condition (Lorig and Holman, 2003). Self-management in hypertensive patients can cover multiple aspects, including but not limited to: blood pressure monitoring, medication adherence, lifestyle management, and symptom management (Glynn et al., 2010). Research consistently reveals that enhancing self-management abilities among chronic disease patients significantly boosts overall health (Allegrante et al., 2019). Effective self-management positively influences hypertension treatment and control, lowering the probability of poor blood pressure regulation (Qu et al., 2019), diminishing complication rates (Dickinson et al., 2006), easing disease burden (Long et al., 2017), and even enhancing quality of life (Stuart-Shor et al., 2012). However, young and middle-aged hypertensive patients in China often fall short in self-management proficiency (Wang and Liu, 2018). Early in hypertension symptomatology, patients frequently underestimate condition severity, leading to inadequate treatment (Wang et al., 2017). Proactive self-management typically arises only upon awareness of severe complications, such as stroke (van Bussel et al., 2019). Given the pivotal role of young and middle-aged adults in the labor force and the profound impact of their health on national well-being, research attention has increasingly focused on understanding self-management levels in hypertensive patients within this age bracket. Thus, comprehensive examination of self-management status among young and middle-aged hypertensive patients in rural communities is imperative.

### 1.1 Literature review

The effectiveness of self-management is contingent upon an array of determinants, encompassing individual health literacy, illness perception, and self-efficacy, among other factors.

#### 1.1.1 Health literacy

Health literacy, a term introduced in the 1970s, embodies the understanding of one’s own health, as well as that of their family and community, encompassing awareness of the factors impinging upon it and the knowledge of how to address these issues effectively. An individual possessing an adequate level of health literacy demonstrates the capacity for assuming responsibility not only for their personal well-being but also for the health of their household and the broader communal environment (McQueen et al., 2007; Sørensen et al., 2012). Numerous studies have consistently shown that individuals with good health literacy are more likely to effectively manage their own health. Shaw et al. (2012) and Murphy et al. (2015) conducted studies that revealed how a lack of health literacy poses a barrier for hypertensive patients seeking to improve their self-management skills. Additionally, Rimando (2015) highlighted the influence of health literacy on patients’ decision-making abilities in self-management (Ishikawa and Yano, 2011). In a study focusing on uncontrolled hypertensive patients, Persell et al. (2020) discovered a significant correlation between lower health literacy and poor self-management across multiple aspects of medication management. The findings collectively underscore the importance of health literacy in shaping the health behaviors and outcomes of individuals with chronic diseases, particularly hypertensive patients. However, it is worth noting that current research has primarily relied on correlational studies. It is crucial to delve into the underlying mechanisms through which health literacy influences self-management.

#### 1.1.2 Illness perception

Illness perception refers to an individual’s subjective experience and understanding of their own health status and illness, making it a vital variable in the realm of chronic disease management (Leysen et al., 2015). Research has indicated that illness perception can influences patients’ coping behaviors (Liu et al., 2021), and addressing negative illness perception can effectively enhance patients’ ability to self-manage their condition (Xiong et al., 2023). Illness perception plays a crucial role in the self-management of hypertension, with strong associations observed between favorable disease control perception and adherence to medication usage, as well as self-monitoring (Lu et al., 2022); treatment control, risk factors, and psychological attributions, among other factors, can influence compliance with self-management recommendations (Chen et al., 2009). Moreover, a significant correlation has been established between health literacy and illness perception (Pérez, 2015; Shiyanbola et al., 2018). Higher levels of health literacy facilitate enhanced understanding of disease prevention, diagnosis, and treatment, thereby fostering a more objective and rational illness perception. Such accurate disease cognition serves to mitigate misconceptions and anxiety surrounding the condition, fostering active patient engagement and bolstering both the willingness and capacity for self-management.

#### 1.1.3 Self-efficacy

Self-efficacy refers to an individual’s belief in their capability to perform a specific behavior and achieve desired outcomes (Xie et al., 2020). High self-efficacy has emerged as a proven facilitator of patients’ self-management. Extensive research suggests a close relationship between self-efficacy, treatment adherence, and health outcomes among hypertensive patients, making it a crucial factor influencing patients’ commitment to self-management (Zimbudzi et al., 2017). Simultaneously, health literacy empowers patients to attain a deeper understanding of their disease and corresponding treatment regimens, thereby augmenting their sense of self-efficacy, the belief in their capacity to effectively manage their condition. Patients with a robust sense of self-efficacy are more inclined to engage in proactive self-management behaviors, such as regular blood pressure monitoring, adhering to prescribed medication regimens, and maintaining a healthy lifestyle. Consequently, enhancing health literacy and self-efficacy fosters improved self-management practices, ultimately contributing to more favorable health outcomes (Warren-Findlow et al., 2012; Dinh and Bonner, 2023).

#### 1.1.4 Health literacy skills framework

Health Literacy Skills Framework, proposed by Squiers et al. (2012), is a comprehensive model that delineates how individuals access, evaluate, and apply health information. The framework comprises four main components: factors influencing the development and utilization of health literacy skills; health-related stimuli; health literacy skills required to comprehend stimuli and perform tasks; and the intermediary relationship between health literacy and health outcomes. The HLS theory integrates individual and environmental factors with health outcomes, with a primary focus on understanding the direct and indirect effects of health literacy on health outcomes. Based on the successful research experience of this model in the field of chronic diseases (Wong et al., 2018; Rueda-Medina et al., 2020), this particular investigation defines hypertension as a health-related stimulus, illness perception as the comprehension of stimuli, self-efficacy as an intermediary factor, and self-management as a critical health behavior linked to positive health outcomes. Through the application of structural equation modeling, the study further examines how health literacy, illness perception, and self-efficacy influence self-management among young and middle-aged rural individuals diagnosed with hypertension.

Building on HLS model and existing research findings, we put forward the following hypotheses.

The first hypothesis suggests that health literacy directly influences an individual’s ability to self-manage their health. The second hypothesis proposes that health literacy indirectly affects self-management by influencing how individuals perceive their illness. The third hypothesis suggests that health literacy indirectly influences self-management through its impact on individuals’ self-efficacy. Treating self-efficacy and illness perception as parallel mediators illuminates how two distinct, empirically validated established psychological pathways, independently influence the association between health literacy and self-management, even if the effect of one variable is weaker or nonsignificant, the other may still exert a significant impact through its unique pathway.

Lastly, the fourth hypothesis posits that health literacy indirectly impacts self-management through both illness perception and self-efficacy. In this pathway, it is hypothesized that illness perception impacts self-efficacy. If patients view their illness as an insurmountable adversity, it may lead to emotional distress which undermines self-efficacy. Conversely, regarding disease in a more positive, adaptive manner as a manageable life challenge could promote an affective state more conducive towards bolstering self-efficacy. This would reinforce patients’ conviction in their own ability to adhere to healthcare professionals’ recommended prescription regimens and lifestyle modifications. This hypothesis finds empirical corroboration in extant research findings (Lau-Walker, 2004; Lau-Walker, 2006; Knowles et al., 2020).

**Figure 1:**
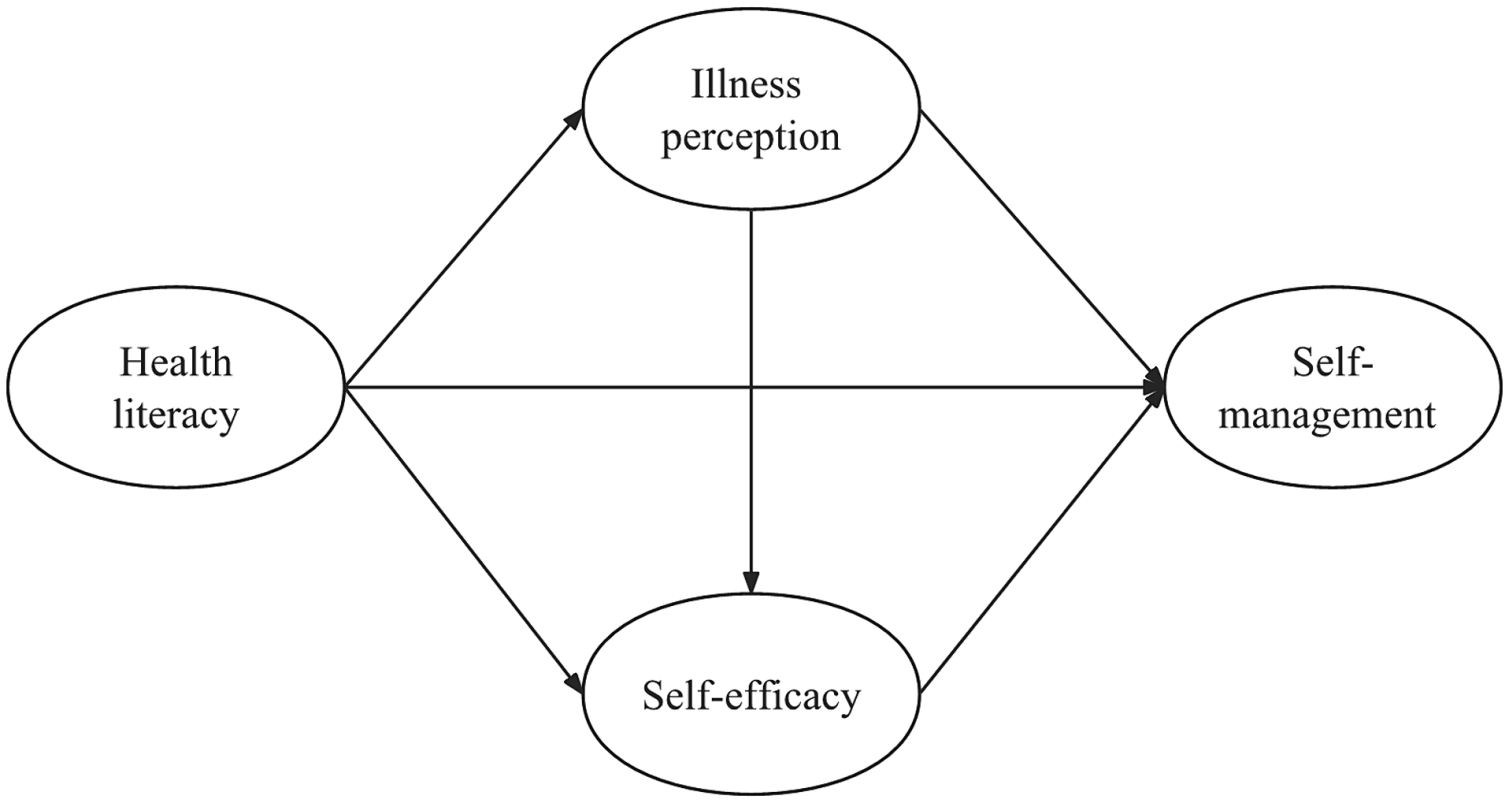
Hypothesized structural equation model.

Overall, we have developed an initial theoretical model (Figure 1).

## 2 Methods

### 2.1 Data sources

A multi-stage, stratified random sampling strategy was employed within Sichuan Province to select the study sample. Notably, Chengdu City, characterized by relatively favorable economic conditions, and three ethnic autonomous prefectures exhibiting relatively impoverished economic circumstances were intentionally excluded from the sampling frame. Among the remaining 17 cities, four were randomly selected: Zigong, Yibin, Mianyang, and Bazhong. Within each of these chosen cities, two villages were randomly picked for questionnaire distribution.

Eligibility for inclusion in the sample was determined according to the following criteria: participants aged between 18 and 59 years, with a clinical diagnosis of hypertension, voluntarily consenting to participate in the study, and providing written informed consent by signing the designated consent form.

Exclusion criteria for the sample comprised the following: individuals suffering from severe mental disorders, confusion, impaired communication, or uncooperative behavior; those with malignant tumors, severe cardiac pathologies, or other grave comorbidities; and patients with secondary hypertension stemming from alternative etiologies.

Data on hypertensive patients were gathered from the family physicians serving each of the sampled villages. These healthcare professionals also played a crucial role in identifying and excluding individuals who failed to meet the established inclusion criteria. A total of 352 questionnaires were administered, out of which 338 were deemed valid, whereas 14 were deemed invalid. This yielded an effective response rate of 96.02%.

### 2.2 Measurement

#### 2.2.1 Health literacy

The measurement of health literacy in this study utilized the Health Literacy Management Scale (HeLMS) (Jordan et al., 2013), which was translated and culturally adapted. It consists of 24 items distributed across four dimensions. Each item is rated on a 5-point Likert scale, with a maximum score of 120. Individuals obtaining higher scores are regarded as possessing higher levels of health literacy, with those scoring above 96 are considered to have a high level. The internal consistency reliability of the scale was assessed using Cronbach’s alpha coefficient, resulting in a value of 0.896.

#### 2.2.2 Illness perception

The measurement of illness perception utilized the Brief Illness Perception Questionnaire (BIPQ) developed by Broadbent et al. (2006). It consists of 9 items distributed across 3 dimensions. The scoring for the first 8 items ranges from 0 to 10, with a maximum total score of 80. Higher scores in patients indicate a greater perceived disease burden. The 9th item is an open-ended question. In this study, the standardized Cronbach’s alpha coefficient for the scale was calculated to be 0.807.

#### 2.2.3 Self-efficacy

The self-efficacy scale used in this study was the Chinese Hypertension Self-Efficacy Scale developed by Ji Shaorong in 2015 (Shaorong, 2018). It includes 4 dimensions and 11 items. Each item is rated on a 0–4 scale, with a maximum total score of 44. An individual’s higher score represents a greater level of confidence in their ability to achieve desired outcomes through their actions. The Cronbach’s alpha coefficient for the self-efficacy scale was calculated as 0.862.

#### 2.2.4 Self-management

The self-management scale utilized in this study was the Chinese Hypertension Self-Management Scale developed by Ning et al. (2015). The scale comprises 21 items distributed across 4 dimensions. Each item is assessed using a 5-point Likert scale, with a maximum score of 105. Individuals with higher scores are considered to possess a higher level of self-management ability. The reliability analysis demonstrated a Cronbach’s alpha coefficient of 0.884.

### 2.3 Statistical analysis

Survey data were entered into IBM Corp.’s SPSS 26.0, with thorough error correction and missing value resolution to ensure accuracy. Comprehensive statistical analysis followed, employing SPSS 26.0, AMOS 26.0, and the processv34 plugin. A structural equation model (SEM) was first established in alignment with the theoretical model in Figure 1, subsequently refined by eliminating insignificant correlations. The bootstrap method was used to validate mediating effects. Model fit was evaluated using multiple indices: Root Mean Square Error of Approximation (RMSEA) < 0.10, Comparative Fit Index (CFI) > 0.90, Goodness-of-Fit Index (GFI) > 0.90, Normed Fit Index (NFI) > 0.90, Incremental Fit Index (IFI) > 0.90, and Tucker-Lewis Index (TLI) > 0.90 (Browne and Cudeck, 1993).

## 3 Results

### 3.1 Patient demographics

This study analyzed a total of 338 valid samples. The age range was 32 to 59 years, with an average age of (51.8 ± 5.9) years. Of the patients, 54.73% were male and 45.27% were female. Among the surveyed samples, the majority (94.67%) were married, 56.21% of the patients had an elementary school education or below, while 39.05% had a junior high school education, indicating a generally low educational level. In terms of residential status, 71.89% of the patients lived together with their spouses, and 13.91% lived with both their spouse and children. More than half of the patients had a course of hypertension of 3–5 years, and 69.82% of the patients had grade 1 hypertension. The self-management score of the sample was 70.45 ± 11.36. Additional details can be found in Table 1.

**Table 1:** Basic demographics of rural young and middle-aged patients with hypertension.

**Variable**	**Overall (*n* = 338)**
	***x*^−^ ± *SD*/*n* (%)**
Age, Mean	51.8 ± 5.9
**Age**	
Aged 18–45	77(22.78)
Aged 46–59	261(77.22)
**Gender**	
Male	185(54.73)
Female	153(45.27)
**Marital status**	
Married	320(94.67)
Single	9(2.66)
Widowed	9(2.66)
**Education**	
Elementary school and below	190(56.21)
Junior high school	132(39.05)
Technical school or high school	13(3.85)
College or above	3(0.89)
**Residential status**	
Living alone	36(10.65)
Living with spouse	243(71.89)
Living with children	6(1.78)
Living with spouse and children	47(13.91)
Other	6(1.78)
**Course of hypertension**	
Less than 3 years	107(31.66)
3–5 years	184(54.44)
6–10 years	29(8.58)
More than 10 years	18(5.33)
**Classification of hypertension**	
Grade 1 (140–159 mmHg/90–99 mmHg)	236(69.82)
Grade 2 (160–170 mmHg/100–109 mmHg)	85(25.15)
Grade 3 (180 mmHg or higher/110 mmHg or higher)	17(5.03)
**Health literacy score**	84.44 ± 0.59
**Illness perception score**	42.32 ± 11.62
**Self-efficacy score**	28.49 ± 6.95
**Self-management score**	70.45 ± 11.36

SD, standard deviation.

### 3.2 Correlation analyses

The correlations among health literacy, illness perception, self-efficacy, and self-management were presented in Table 2. As the results demonstrate, significant correlations exist among all four variables, with health literacy showing positive associations with self-efficacy (*r* = 0.534, *p* < 0.01) and self-management (*r* = 0.561, *p* < 0.01). Self-efficacy, in turn, is also positively correlated with self-management (*r* = 0.594, *p* < 0.01). Conversely, illness perception exhibits negative correlations with health literacy (*r* = −0.193, *p* < 0.01), self-efficacy (*r* = −0.208, *p* < 0.01), and self-management (*r* = −0.295, *p* < 0.01).

**Table 2:** Correlation matrix of health literacy, illness perception, self-efficacy, and self-management.

**Variables**	**Health literacy**	**Illness perception**	**Self-efficacy**	**Self-management**
Health literacy	–			
Illness perception	−0.193**	–		
Self-efficacy	0.534**	−0.208**	–	
Self-management	0.561**	−0.295**	0.594**	–

**p < 0.01.

### 3.3 Structural equation modeling

**Figure 2:**
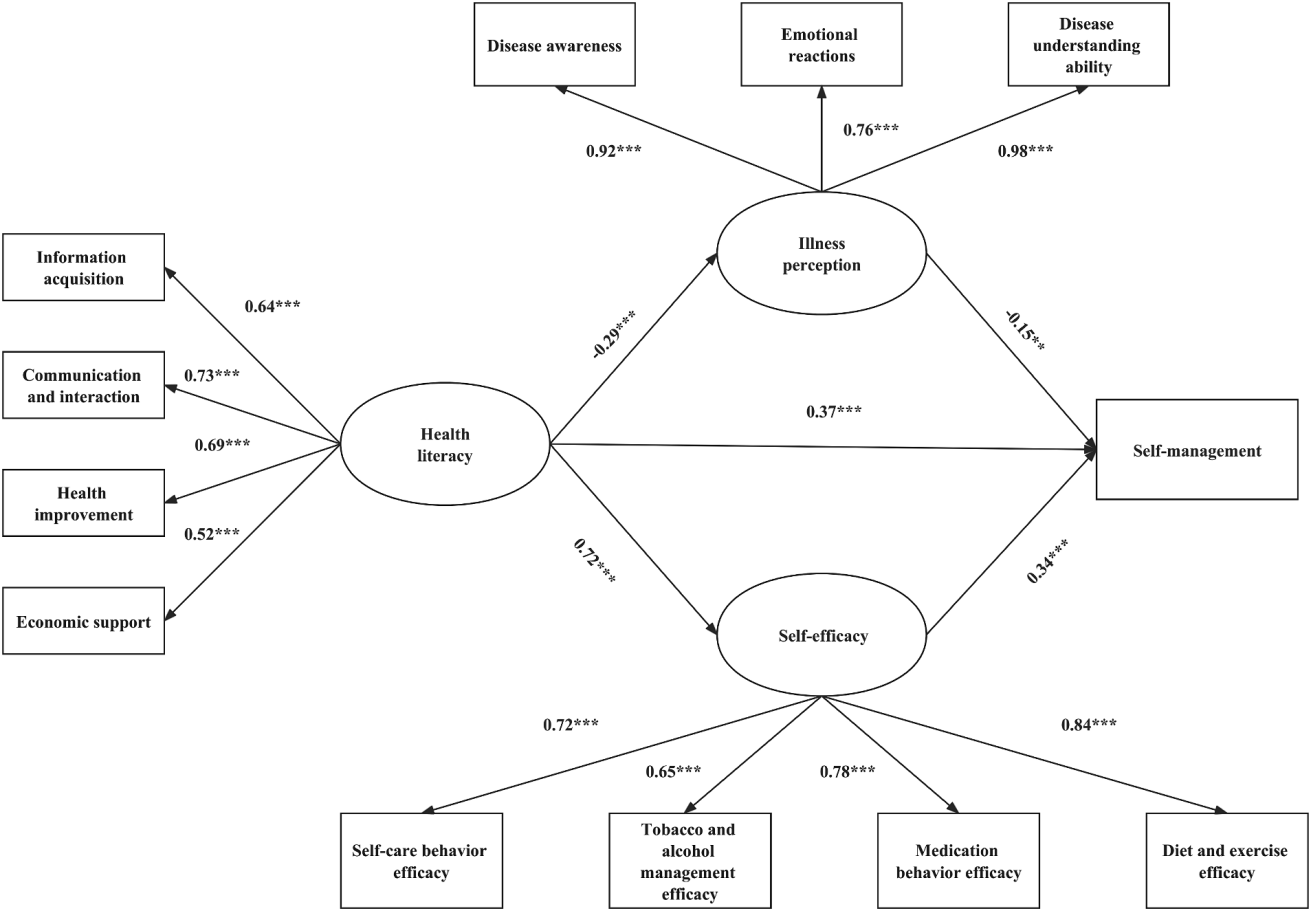
Final model with standardized path coefficients and significant level. Fit statistics: Comparative Fit Index (CFI) = 0.91; Tucker–Lewis Index (TLI) = 0.91; Root Mean Square Error of Approximation (RMSEA) = 0.095. ***p < 0.001; **p < 0.01; *p < 0.05.

A structural equation model was preliminarily established based on the theoretical framework. After confirmatory factor analysis, the model fit indices were as follows: GFI = 0.90, NFI = 0.90, IFI = 0.92, TLI = 0.89, CFI = 0.92, RMSEA = 0.10. However, the path from illness perception to self-efficacy was not significant (*p* = 0.38) and was subsequently removed from the model. The final model, shown in Figure 2, had improved fit indices: GFI = 0.91, NFI = 0.91, IFI = 0.93, TLI = 0.91, CFI = 0.93, RMSEA = 0.095. This indicated a good fit between the model and the data, where all path coefficients and factor loadings were significant in the model.

According to the final model results shown in Table 3, both health literacy and self-efficacy demonstrated a positive association with self-management (std. *β* = 0.372, *p* < 0.001; std. *β* = 0.344, *p* < 0.001). Conversely, there was a negative correlation between illness perception and self-management (std. *β* = −0.146, *p* < 0.01). Moreover, health literacy was found to be negatively associated with illness perception (std. *β* = −0.291, *p* < 0.001) while positively associated with self-efficacy (std. *β* = 0.717, *p* < 0.001).

**Table 3:** Path coefficient test for the final SEM model.

**Independent variable**	**Dependent variable**	***β***	***S.E.***	***C.R.***
Health literacy	Illness perception	−0.291***	0.686	−4.261
Health literacy	Self-efficacy	0.717***	0.272	7.703
Health literacy	Self-management	0.372***	1.504	4.158
Illness perception	Self-management	−0.146**	0.074	−3.305
Self-efficacy	Self-management	0.344***	0.470	4.197

β, standardized path coefficient; S.E., standard error; C.R., critical ratio. ***P < 0.001; **P < 0.01.

### 3.4 Mediation analysis

The analysis in Table 4 examined the mediating effect of illness perception and self-efficacy on the relationship between health literacy and self-management, using the Bootstrap method. The results indicate that when considering illness perception as a mediator, the direct effect of health literacy on self-management is 0.561 (SE = 0.048, 95% CI [0.467, 0.656], *p* < 0.01), accounting for 93.32% of the total effect. Its indirect effect is 0.040 (SE = 0.014, 95% CI [0.016, 0.069], *p* < 0.01), accounting for 6.68% of the total effect. In the pathway where self-efficacy serves as a mediator, the direct effect of health literacy on self-management is 0.365 (SE = 0.052, 95% CI [0.263, 0.467], *p* < 0.01), making up 60.69% of the total effect, while its indirect effect is 0.236 (SE = 0.037, 95% CI [0.169, 0.313], *p* < 0.01), contributing 39.31% of the total effect. The overall indirect effect is 0.276, and the total effect through both mediators were identical at 0.602 (illness perception: SE = 0.049, 95% CI [0.506, 0.697], *p* < 0.01; self-efficacy: SE = 0.048, 95% CI [0.506, 0.697], *p* < 0.01).

**Table 4:** Mediation effect with illness perception/self-efficacy as the mediating variable.

**Mediating variable**	**Path (Health literacy → Self-management)**	**Effect value**	***S.E.***	**LLCI**	**ULCI**	***P***-value	**Effect percentage (%)**
Illness perception	Total effect	0.602	0.049	0.506	0.697	<0.01	–
	Direct effect	0.561	0.048	0.467	0.656	<0.01	93.32%
	Indirect effect	0.040	0.014	0.016	0.069	<0.01	6.68%
Self-efficacy	Total effect	0.602	0.048	0.506	0.697	<0.01	-
	Direct effect	0.365	0.052	0.263	0.467	<0.01	60.69%
	Indirect effect	0.236	0.037	0.169	0.313	<0.01	39.31%

S.E., standard error; LLCI, lower limit of confidence interval; ULCI, upper limit of confidence interval.

## 4 Discussion

The present investigation confirms that health literacy and self-efficacy are critical predictors of improved self-management, whereas illness perception is negatively associated with self-management. Additionally, illness perception and self-efficacy partially mediate the relationship between health literacy and self-management. These findings highlight the importance of mitigating negative illness perceptions and enhancing health literacy and self-efficacy to foster effective self-management among hypertensive populations.

This study examined young and middle-aged hypertensive patients in rural areas and discovered that their self-management scores (70.45 ± 11.36) were inferior to those reported for young and middle-aged hypertensive patients (74.94 ± 3.12) in prior research encompassing both urban and rural inhabitants (Hongchun, 2021). The scores were also marginally below those documented previously among community-dwelling elderly hypertension patients (71.52 ± 8.1) in a study concentrated on urban seniors (Yuan et al., 2019). There could be several reasons for this: Firstly, 56.21% of the patients in this study had an education level of elementary school or below, indicating a lower educational background and limited understanding of health-related knowledge regarding chronic diseases such as hypertension, resulting in weaker self-management awareness. Secondly, rural areas often suffer from a lack of medical resources, incomplete chronic disease management teams, and an inadequate social support system, which contributes to a lack of attention and support for patients. Lastly, the natural environment and infrastructure in rural areas differ from urban areas, with a lack of exercise facilities and a conducive health atmosphere, which limits the improvement of patients’ self-management levels.

The results of the structural equation modeling and mediation analysis further validate the theoretical model within the Health Literacy Skills Framework. Specifically, this study examines the intricate relationships between health literacy skills, stimulus comprehension, mediating factors, and health behaviors. Firstly, in alignment with previous research findings (Ishikawa and Yano, 2011; Rimando, 2015; Persell et al., 2020), rural young and middle-aged adults with robust health literacy skills and high blood pressure demonstrate active engagement in self-management practices. A heightened level of health literacy equips individuals with essential tools and knowledge, enabling them to proactively manage their health. These individuals possess a comprehensive understanding of health-related information and resources, including the ability to comprehend medical instructions, navigate complex healthcare systems, make informed lifestyle choices, and effectively communicate with healthcare professionals (Nutbeam and Lloyd, 2021). Consequently, they are empowered to make informed decisions and take appropriate actions to safeguard their well-being.

Additionally, patients exhibiting superior health literacy skills demonstrate greater attentiveness and sense of importance towards their health status. They are more likely to accurately perceive and interpret symptoms, signs, and information relevant to their condition, comprehend potential risks and consequences, and possess the knowledge and capabilities to take appropriate actions to manage and mitigate these risks. Thereby, they can better cope with the negative emotional responses triggered by disease stimuli and form positive cognition, alongside adopting proactive self-management measures. These findings provide further support to prior evidence that both health literacy and illness perception play a role in impacting self-management behaviors (Pérez, 2015; Ajuwon and Insel, 2022).

On the other hand, individuals possessing competent health literacy have a clearer recognition of their health condition and a more profound grasp and mastery of health knowledge and skills. This enhances their confidence to execute appropriate behaviors and regulate disease progression, thereby amplifying their self-efficacy. This encourages patients to better implement health management know-how in practice, hence strengthening self-management capabilities, alleviating negative impacts, harnessing health risks, and ameliorating blood pressure control (Suarilah and Lin, 2022). Parallelly, research has exhibited self-efficacy mediating the association between eHealth literacy and self-care to some degree among chronic disease populations (Wu et al., 2022).

This study furnishes valuable policy insights for the prevention and management of hypertension among young and middle-aged populations residing in rural contexts. Primarily, there is a pressing need to fortify their self-management capabilities. One strategic avenue to accomplish this is through the widespread promotion and adoption of intelligent blood pressure monitoring devices, thereby augmenting the frequency with which patients monitor their blood pressure. Secondly, a holistic approach should be adopted to elevate their health literacy levels. This multifaceted effort may encompass intensified health education initiatives, the promotion of healthful lifestyle habits, and advocacy for the utilization of innovative health monitoring technologies. Thirdly, concerted efforts should be directed towards mitigating negative disease perceptions held by this population. This can be effectively achieved by disseminating relevant health knowledge and evidence-based treatment modalities, thereby bolstering their confidence in the efficacy of therapeutic interventions. Lastly, targeted support should be provided to enhance their self-efficacy. For instance, proactively showcasing success stories of hypertension control among peers can serve to reinforce their capacity for autonomous decision-making and self-regulation, ultimately fostering a heightened sense of self-efficacy in managing their condition.

However, it is important to acknowledge the limitations of this study. Firstly, the survey focused only on young and middle-aged individuals with hypertension in eight rural villages from four prefecture-level cities in Sichuan province. It is possible that there are regional variations in terms of cultural, economic, and other factors that could influence the findings. To obtain more comprehensive results, future research should include a larger geographic area to ensure a more representative sample. Additionally, this study was cross-sectional in nature, which means that it captured a snapshot of the participants’ health literacy, illness perception, self-efficacy, and self-management at a specific point in time. It is unclear whether these relationships will remain constant or change over time. Therefore, conducting longitudinal studies in the future would be beneficial to better understand the long-term dynamics of these variables. Finally, this study was unable to conduct a meticulous subgroup analysis on different age groups in the application of structural equation modeling. Future researchers should develop more targeted study schemes and ensure sufficient sample sizes, in order to realize independent and in-depth discussions on subgroups of different ages, thus making up for this deficiency.

## 5 Conclusion

Young and middle-aged individuals with hypertension in rural areas exhibit a relatively low level of self-management. Health literacy emerges as a potent positive driver of self-management, with illness perception and self-efficacy functioning as parallel mediators between health literacy and self-management. It is of paramount importance to intensify attention on fostering self-management behaviors among this demographic, augment their health literacy, mitigate negative illness perceptions, and bolster their self-efficacy.

## Data availability statement

The raw data supporting the conclusions of this article will be made available by the authors, without undue reservation.

## Ethics statement

The studies involving humans were approved by Methods in this study were reviewed and approved by the Institutional Review Board (IRB) of Huazhong University of Science and Technology Tongji Medical College (No. 2023S104) and were in accordance with the 1964 Helsinki declaration and its later amendments, or comparable ethical standards. The studies were conducted in accordance with the local legislation and institutional requirements. Written informed consent for participation was not required from the participants or the participants’ legal guardians/next of kin in accordance with the national legislation and institutional requirements.

## Author contributions

YL: Conceptualization, Data curation, Formal analysis, Investigation, Methodology, Project administration, Supervision, Writing – original draft, Writing – review & editing. FJ: Data curation, Investigation, Methodology, Project administration, Software, Supervision, Validation, Visualization, Writing – original draft, Writing – review & editing. MZ: Formal analysis, Methodology, Project administration, Software, Supervision, Validation, Writing – original draft, Writing – review & editing. HN: Methodology, Software, Supervision, Writing – review & editing. JC: Methodology, Software, Supervision, Writing – review & editing. SD: Methodology, Software, Supervision, Writing – review & editing. HC: Methodology, Software, Supervision, Writing – review & editing. HWa: Methodology, Software, Supervision, Writing – review & editing. LG: Methodology, Software, Supervision, Writing – review & editing. FR: Methodology, Software, Supervision, Writing – review & editing. HWu: Methodology, Software, Supervision, Writing – review & editing.
